# Characterization and In Vitro Antioxidant and Anti-Inflammatory Activities of Ginsenosides Extracted from Forest-Grown Wild *Panax quinquefolius* L.

**DOI:** 10.3390/foods12234316

**Published:** 2023-11-29

**Authors:** Yang Yang, Shan Xu, Kemeng Yang, Yuning Sun, Ruirui Yang, Yanan Hu, Guijie Chen, Huimei Cai

**Affiliations:** 1 School of Tea and Food Science & Technology, Anhui Agricultural University, Hefei 230036, China; ahhsyy0512@163.com (Y.Y.); annongdaxushan@sina.com (S.X.); 15282684556@163.com (K.Y.); 13205612767@163.com (Y.S.); yrr_ahau@163.com (R.Y.); 18409573505@163.com (Y.H.); guijiechen@ahau.edu.cn (G.C.); 2 State Key Laboratory of Tea Plant Biology and Utilization, Anhui Agricultural University, Hefei 230036, China

**Keywords:** functional food, plant natural products, extraction methods, bioactive compounds, anti-inflammatory

## Abstract

American ginseng (*Panax quinquefolius* L.) is known for its health benefits, which are attributed to various terpenoids. However, the specific composition and activities of these terpenoids in forest-grown wild American ginseng remain understudied. This study aimed to characterize the terpenoid composition, particularly triterpene saponins, in forest-grown wild American ginseng. The analysis revealed that triterpene saponins, notably American ginseng ginsenosides (AGGs), are the predominant active components, as identified through LC-MS/MS and HPLC. A subsequent in vitro evaluation of AGGs showcased their potent antioxidant capabilities, displaying the dose-dependent scavenging of free radicals and reducing agents. Moreover, AGGs demonstrated efficacy in reducing oxidative injury and intracellular ROS levels in RAW 264.7 macrophages treated with H_2_O_2_. In addition to their antioxidant properties, AGGs exhibited anti-inflammatory effects, significantly inhibiting NO and inflammatory substance production in lipopolysaccharide-treated RAW 264.7 macrophages. These findings highlight the potential of AGG-rich forest-grown wild American ginseng as a functional food with promising implications for improving human health.

## 1. Introduction

In recent years, there has been an increasing fascination with health-enhancing products [1]. Ginseng and American ginseng are precious medicinal materials and perennial herbs of the ginseng genus in the family *Anacanthaceae*. American ginseng (*Panax quinquefolius* L.), in particular, has gained widespread attention in the international market due to its long history of cultivation and medicinal use [2]. Moreover, studies have confirmed that Chinese ginseng is of higher quality than Korean ginseng, has stronger in vitro antioxidant activity, and is more resistant to rust and rot, as well as having better root morphology [3]. The health-promoting functions of American ginseng, such as its effects on the cardio-cerebrovascular and immune systems, have been widely reported [4]. In recent years, the understory planting system, as a novel plant method, has received much attention due to its various advantages. Lan et al. [5] reported that cultivating forest-grown wild American ginseng can help reduce costs, increase market prices, improve ecological health, and maintain biodiversity. As a result, forest-grown wild American ginseng has gained widespread acceptance in different regions worldwide. Forest-grown wild *P. quinquefolius* possesses distinctive characteristics that distinguish it from other ginseng varieties. The cultivation environment of ginseng significantly influences its phytochemical composition. This study aims to emphasize the unique qualities of forest-grown ginseng, especially in a natural, wild forest setting, which can lead to a distinct chemical profile in comparison to commercially cultivated varieties. The specific environmental conditions encompassing soil composition, climate, and geographical locations contribute to variations in ginsenoside composition. These variations may result in a different balance of ginsenosides and other bioactive compounds, potentially imparting specific antioxidant, anti-inflammatory, or other medicinal properties.

Terpenoids, flavonoids, and phenolics serve as the primary bioactive ingredients of American ginseng. Ginsenosides, which are triterpene saponins and the predominant ingredient in American ginseng, have been demonstrated to have unique medicinal effects, encompassing antibacterial, anti-inflammatory, and anti-cancer characteristics, as well as the ability to treat chronic lung diseases and alleviate symptoms of depression [6]. Ginsenoside Rc has been indicated to reduce myocardial ischemic injury through antioxidant and anti-inflammatory mechanisms, while Rg1 has been shown to mitigate manifestations related to Alzheimer’s disease [7]. Furthermore, Rg3 has been demonstrated as a candidate for treating oxidative-stress- and inflammation-related diseases [8]. Other studies have demonstrated that ginsenosides, including Rb1, Rh2, and Re, can decrease inflammatory cytokine levels and suppress inflammatory mediator activation by inhibiting macrophage-induced inflammatory responses [9]. Flavonoids and phenolics are also abundant in American ginseng and have been found to be the primary sources of the herb’s strong antioxidant activity [10]. Specifically, studies have shown that the predominant factors contributing to the potent antioxidant activity of extracts from ginseng leaves are flavonoids and phenolics [11]. As a result, the application of these components in the clinical setting and daily life has become a major focus for the development and advancement of human nutrition and health.

American ginseng is gaining increasing attention as both a medicinal herb and a food plant. However, the composition and health-promoting effects of bioactive compounds in forest-grown wild American ginseng have received little attention. Until now, the majority of research in this area has primarily focused on cultivated American ginseng and its growth patterns under various environmental conditions. However, there has been relatively limited investigation into wild ginseng and its constituent components within its natural forest habitat. Consequently, our study aims to fill this gap in knowledge. Additionally, our research encompasses a thorough evaluation of the antioxidant and anti-inflammatory properties of the extracted ginsenosides. These biological activities hold great significance in the realm of medicinal plants. By expanding the understanding of the bioactive characteristics of ginsenosides, our study offers new evidence for their potential medicinal value. In this study, the main active components in forest-grown wild American ginseng were extracted and then identified. The in vitro antioxidant effects of the active components were assessed by measuring their free-radical-scavenging activity, and their protective effects were evaluated based on their ability to protect cells from oxidative damage induced by H_2_O_2_. Additionally, the anti-inflammatory potential of active ingredients was evaluated by assessing their effects on RAW 264.7 macrophages induced by LPS. Our study hypothesizes that wild *P. quinquefolius* possesses unique ginsenosides due to its natural habitat, potentially exhibiting superior antioxidant and anti-inflammatory activities. By exploring its distinct chemical composition and potential medicinal implications, we aim to fill the knowledge gap concerning the growth of wild American ginseng and provide comprehensive information on its antioxidant and anti-inflammatory activities. This research will offer essential reference points for the further exploration and potential medicinal applications of wild ginseng.

## 2. Materials and Methods

### 2.1. Materials and Chemicals

American ginseng cultivated in Lu’an, Anhui Province (31°01′–32°40′ N, 115°20′–117°14′ E), was collected in September 2020 from the same forest type (10 m × 10 m), and samples of wild American ginseng with the same growth year and similar plant growth were selected for the experiment, sourced from Anhui Luyuan Wild American Ginseng Co., Ltd. (Hefei, China). Standard ginsenosides Rg1 (≥98%), Re (≥98%), Rf (≥98%), Rb1 (≥98%), Rc (≥98%), Rg2 (≥98%), Rb2 (≥98%), Rd (≥98%), F2 (≥98%), and Rg3 (≥98%) were acquired from Sigma-Aldrich (Shanghai, China). RAW 264.7 macrophages were cultured following established methods [12]. Aiammonium salt (ABTS, ≥98%), phenazine methosulfate (PMS, ≥98%), 1,1-diphenyl-2-picrylhydrazyl (DPPH, ≥98%), and nitroblue tetrazolium (NBT, ≥98%) were procured from Sigma Chemical Co. (St. Louis, MO, USA). Cell Counting Kit-8 (CCK-8, C0037) for cell proliferation and cytotoxicity detection, NO (nitric oxide) assay kit (S0012S; sensitivity: 0.2 µmol/L), and ROS (reactive oxygen species) assay kit (S0033S) were procured from Beyotime Co., Ltd. (Shanghai, China). A TNF-α (tumor necrosis factor-alpha) assay kit (MB-28668A), IL-6 (interleukin-6) assay kit (MB-2899A), and IL-1β (interleukin-1 beta) assay kit (MB-2776A; sensitivity: 4 pg/mL) were obtained from Mei Biao Biological Technology Co., Ltd. (Jiangsu, China). Ethanol, ascorbic acid (Vc), NaNO_2_, AlCl_3_, NaOH, methanol, acetonitrile, acetic acid, pyrogallic acid, FeSO_4_, H_2_O_2_, potassium persulfate, and ortho-hydroxybenzoic acid were of analytical grade and purchased from Sinopharm Chemical Reagent Co., Ltd. (Shanghai, China).

### 2.2. Extraction of Active Components of Forest-Grown Wild American Ginseng

Total ginsenosides were extracted using the established method reported by Chen et al. [13] with several alterations. Initially, forest-grown wild American ginseng was sliced into approximately 10 cm pieces and dried in the oven at 60 °C, ensuring that they reached a constant weight, and then pulverized using a muller and collected through a 40-mesh sieve. The extraction of total ginsenosides involved the following steps: 1.0 g of the powdered specimen was introduced into 100.0 mL of 50% methanol and subjected to sonication (KH5200DE, Hechao Ultrasonic Instruments Co., Ltd., Kunshan, China) for 30 min to extract ginsenosides (conditions: power, 250 W; frequency, 45 kHz; time, 30 min). After being filtered, evaporated (40 °C, 200 rpm), and lyophilized (the vacuum was maintained at about 20 pa for 48 h), the raw extract was dissolved in distilled water and then loaded on D101 macroporous resin, and further elution was performed sequentially using distilled water and 0.2% sodium hydroxide. The fraction eluted by 50% methanol was collected, evaporated, and lyophilized to afford crude AGGs. The ginsenoside content in the AGG fraction was quantified using the reported approach, with Re used as the standard [14].

### 2.3. Composition of AGGs Evaluated by UPLC-QTOF-MS/MS

The composition of AGGs was examined using UPLC-QTOF-MS/MS following the established approach described by Chen et al. [13], with some alterations. In brief, an ACQUITY UPLC HSS T3 column (2.1 mm × 100 mm, 1.8 µm) was utilized for separation. The column oven temperature was 30 °C, and the auto-sampler temperature was 15 °C. The mobile phase comprised two components: mobile phase A, water (0.1% formic acid), and mobile phase B, acetonitrile (0.1% formic acid). Gradient elution was utilized, with the ratio of mobile phase B beginning with 10% and concluding with 100% in 24 min (2.0–26.0 min), remaining at 100% for 3 min (26.0–29.0 min), decreasing back to 10% in 0.1 min (29.0–29.1 min), and remaining at 10% for 2.9 min (29.1–32.0 min). The rate of flow was adjusted to 0.40 mL/min, and the injection volume was 5 µL. A mass spectrometry (MS) analysis was conducted by employing an Agilent 1290 II UPLC system combined with an Agilent 6545 Q-TOF LC/MS instrument, which was outfitted with an electrospray ionization (ESI) source. The MS results were obtained in negative ion mode, covering the *m*/*z* range of 100–1500 Da. The cone voltage was adjusted to 40 V, while the voltage of the capillary was adjusted to 2.6 kV. The source temperature was 150 °C, while the desolvation temperature was optimized to 400 °C. The flow rate of cone gas was optimized to 50 L/h, while the flow rate of desolvation gas was optimized to 800 L/h. To ensure mass accuracy and reproducibility, a reference (*m*/*z* 554.2615 (ESI^−^) lock spray with leucine-enkephalin (200 pg/mL, 10 μL/min) was utilized. During the acquisition process, data were collected in continuous mode for screening analysis and in mass center mode for metabolomics analysis. Data were analyzed using XCMSplus software (version 03.03).

### 2.4. Contents of AGGs Analyzed by HPLC

The contents of AGGs were analyzed by HPLC according to previous work [15]. In brief, 100 mg of lyophilized AGGs was solubilized in 2 mL of 70% aqueous methanol (*v*/*v*) for 2 h. After centrifugation (12,000 rpm for 15 min) and filtration using a 0.22 μm filter, the prepared solutions were analyzed by HPLC. The HPLC analysis was conducted utilizing a Waters XBribge^®^ (Waters, Shanghai, China) C18 column (4.6 × 250 mm, 5 μm) set to a temperature of 35 °C, and the injection volume was 10 μL at a flow rate of 1.0 mL/min (phase A was water, and phase B was acetonitrile). The solvent system utilized for the analysis comprised water and acetonitrile, and a gradient elution method was employed, with the proportion of acetonitrile increasing from 21% to 85% over a period of 52 min, after which the solvent composition was returned to the initial ratio of 79:21 *v*/*v* at 60.0 min. In addition, 10 standards were prepared as corresponding standard working solutions for Rg1, Re, Rf, Rb1, Rc, Rg2, Rb2, Rd, F2, and Rg3 in 70% methanol.

### 2.5. In Vitro Antioxidant Activity Analysis

#### 2.5.1. Assay of Scavenging Activity on DPPH Free Radicals

The activity of scavenging DPPH free radicals was examined according to the established approach [16] with alterations. Concisely, solutions with concentrations of 200, 400, 600, 800, and 1000 μg/mL were dispensed into a 96-well plate (50 μL/well); in each well, 100 μL of deionized water and 25 μL of methanolic DPPH solution (0.4 mM) were introduced and thoroughly mixed. After being incubated in the absence of light at 25 °C for 30 min, the measurement of absorbance (Abs) was performed at a wavelength of 517 nm. Ascorbic acid (V_C_) served as the positive control. The capacity of DPPH free radical scavenging was determined using the following formula:

DPPH radical scavenging activity (%) = [1 − (Abs_1_ − Abs_2_)/Abs_0_] × 100

Abs_0_ represents the Abs of the control (water instead of sample), Abs_1_ represents the Abs of the sample or V_C_, and Abs_2_ represents the Abs of the sample under identical conditions to Abs_1_ but with methanol instead of DPPH solution.

#### 2.5.2. Assay of Anion-Scavenging Activity on Superoxide Radicals

The anion-scavenging potential of AGGs on superoxide radicals was evaluated by employing a previously reported method with minor alterations [17]. Briefly, 50 μL of prepared solutions with concentrations of 200, 400, 600, 800, and 1000 μg/mL was combined with 50 μL of PMS solution (60 μM), 50 μL of NADH solution (156 μM), and 50 μL of NBT solution (156 μM). After a 5 min incubation at 25 °C, the measurement of Abs was performed at a wavelength of 560 nm. V_C_ served as the positive control. The superoxide-radical-scavenging activity of the samples was determined using the following formula:

Superoxide-radical-scavenging activity (%) = [1 − (Abs_1_ − Abs_2_)/Abs_0_] × 100

Abs_0_ represents the Abs of the control (water instead of sample), Abs_1_ represents the Abs of the sample or V_C_, and Abs_2_ represents the Abs of the sample under identical conditions to Abs_1_ but with 0.1 M phosphate buffer instead of NBT solution.

#### 2.5.3. Assay of Scavenging Activity on ABTS Radicals

The ABTS-radical-scavenging potential of AGGs was assessed following the approach described by [18] with minor alterations. Concisely, the ABTS solution was prepared by combining potassium persulfate (K_2_S_2_O_8_, 4.95 mM) with ABTS (7.0 mM) at ambient temperature and in the absence of light for a duration of 12 h. The ABTS solution was diluted using 0.2 M PBS (pH 7.4), and the Abs was read at 734 nm. Subsequently, 20 μL of the prepared sample was combined with 200 μL of ABTS solution, and Abs at 734 nm was determined following a 6 min incubation at room temperature. V_C_ was employed as the positive control. The ABTS-radical-scavenging capacity of the samples was determined using the provided formula:

ABTS-free-radical-scavenging activity (%) = [1 − (Abs_1_ − Abs_2_)/Abs_0_] × 100

Abs_0_ represents the Abs of the control (water instead of sample), Abs_1_ represents the Abs of the sample or V_C_, and Abs_2_ represents the Abs of the sample (PBS instead of ABTS).

#### 2.5.4. Assay of Ferric-Reducing Antioxidant Potential (FRAP)

The assay of FRAP was conducted following the approach previously described with slight alterations [19]. FRAP reagent was prepared by appropriately proportioning (10:1:1, *v*/*v*/*v*) and mixing 0.3 M acetate buffer (pH 3.6), 10 mM TPTZ (tripyridyltriazine), and ferric chloride. Subsequently, the FRAP reagent (200 µL) was combined with 20 µL of the prepared sample liquid; following a 10 min incubation at 25 °C, the reaction mixture was subjected to Abs measurement at 593 nm.

### 2.6. Antioxidant and Anti-Inflammatory Activities of AGGs on RAW 264.7 Macrophages

#### 2.6.1. Cytotoxicity of AGGs

Using a previously reported method with some modifications [20], the toxic effects of AGGs on macrophages were assessed. Concisely, the culturing of RAW 264.7 macrophages was conducted in a humidified 5% CO_2_ incubator at 37 °C, using DMEM (10% fetal bovine serum (FBS)), 100 U/mL penicillin, and 100 U/mL streptomycin. Subsequently, 200 µL of RAW 264.7 macrophages at a concentration of 1 × 10^5^ cells/mL was added to 96-well plates and incubated for a duration of 24 h. The old medium was replaced with 200 µL of fresh medium containing different concentrations of samples ranging from 50 to 1600 µg/mL. After an additional incubation period of 24 h, 10 µL of CCK-8 solution was transferred to each well, followed by further incubation for 1 h in the absence of light. Finally, the Abs of the supernatant in each well was examined at 450 nm using a microplate reader (Multiskan MK3, Thermo Fisher Scientific, Waltham, MA, USA) in order to evaluate the toxicity of AGGs. DMEM without dissolved ginsenosides served as the control.

#### 2.6.2. The Protective Effect of AGGs on H_2_O_2_-Induced Oxidative Injury

The protective potential of AGGs against oxidative damage induced by H_2_O_2_ was examined following the established method [20]. Concisely, 200 µL of RAW 264.7 macrophages at a concentration of 1 × 10^5^ cells/mL was added to 96-well plates, followed by incubation for 24 h. Subsequently, 200 µL of fresh medium with AGGs at concentrations ranging from 50 to 400 µg/mL was added to replace the old medium. After another 24 h, the old medium was replaced with fresh DMEM (200 μM H_2_O_2_). After co-stimulation for 2 h, the determination of cell viability was conducted by employing the CCK-8 assay, as described previously.

#### 2.6.3. Effects of AGGs on Intracellular ROS of RAW 264.7 Macrophages

The intracellular reactive oxygen species (ROS)-scavenging potential of AGGs was determined by employing the fluorescent probe DCFH-DA following the established method [21]. Concisely, RAW 264.7 macrophages were grown in glass-bottomed confocal microscope dishes (15 mm diameter, 5 × 10^5^ cells per dish) for 24 h. Afterward, each well was pretreated with diverse concentrations of AGGs (50, 100, 200, and 400 μg/mL) for 2 h, after which cells were co-stimulated with H_2_O_2_ at a concentration of 200 μM for 2 h. Cells treated without H_2_O_2_ and with H_2_O_2_ alone served as a blank control and a positive control, respectively. Following that, the cells were subjected to three washes with PBS to eliminate suspended dead cells, and the cells were stained with 20 μM DCFH-DA (dissolved in serum-free DMEM) for 30 min and incubated at 37 °C in the absence of light. After incubation, the supernatant liquid was discarded, and the cells were subjected to three washes with PBS again to remove the excessive DCFH-DA. Finally, the ROS levels were measured by laser confocal scanning microscopy. The excitation (Ex) and emission (Em) wavelengths were 488 nm and 528 nm.

#### 2.6.4. Anti-Inflammatory Activity of AGGs in LPS-Induced RAW 264.7 Macrophages

The anti-inflammatory effects of AGGs on RAW 264.7 macrophages stimulated by LPS were tested by employing a previously reported method with minor modifications [1]. In brief, RAW 264.7 macrophages were cultured at a density of 2 × 10^4^ cells/mL and then classified into five groups: control, LPS-stimulated model group, and AGG intervention groups (6.25, 12.5, and 25 µg/mL). The control group was cultured using DMEM, while the LPS-stimulated model group was cultured using 1 µg/mL LPS for 24 h. The AGG intervention groups were pretreated with DMEM solutions containing 6.25, 12.5, and 25 µg/mL of AGGs for 2 h, followed by stimulation using 1 µg/mL LPS for another 24 h. Lastly, the supernatants of RAW 264.7 macrophages were used to evaluate the levels of NO, TNF-α, IL-6, and IL-1β, according to the instructions of the corresponding kits.

### 2.7. Statistical Analysis

The data were expressed as the mean ± standard deviation, with triple repeats. One-way analysis of variance (ANOVA) with the Tukey test was employed for statistical analysis. Before statistical analysis, the key assumptions for ANOVA, including normality and homogeneity of variance, were checked. Significance was determined using IBM SPSS Statistics 26. Statistical significance was defined as a value of *p* < 0.05. Statistical plots were generated using origin 2022.

## 3. Results and Discussion

### 3.1. Identification of Terpenoids in Panax quinquefolius *L.* by LC-MS

Terpenoids are recognized as the primary bioactive constituents in *P. quinquefolius*. In the present work, the terpenoids were extracted and preliminarily purified from *P. quinquefolius*, and their purity was 77.3 ± 3.56%. Nonetheless, due to the abundance of terpenoids in *P. quinquefolius*, the separation and identification of these compounds pose a significant challenge. Thus, the composition of terpenoids was examined by UPLC-QTOF-MS/MS. The categories and percentages of terpenoids are shown in Figure S1. It was found that 75.8% of terpenoids were triterpene saponins. A lot of previous work has shown that AGGs, belonging to triterpene saponins, may be the key compounds contributing to the health-promoting functions of *P. quinquefolius*.

**Figure 1:**
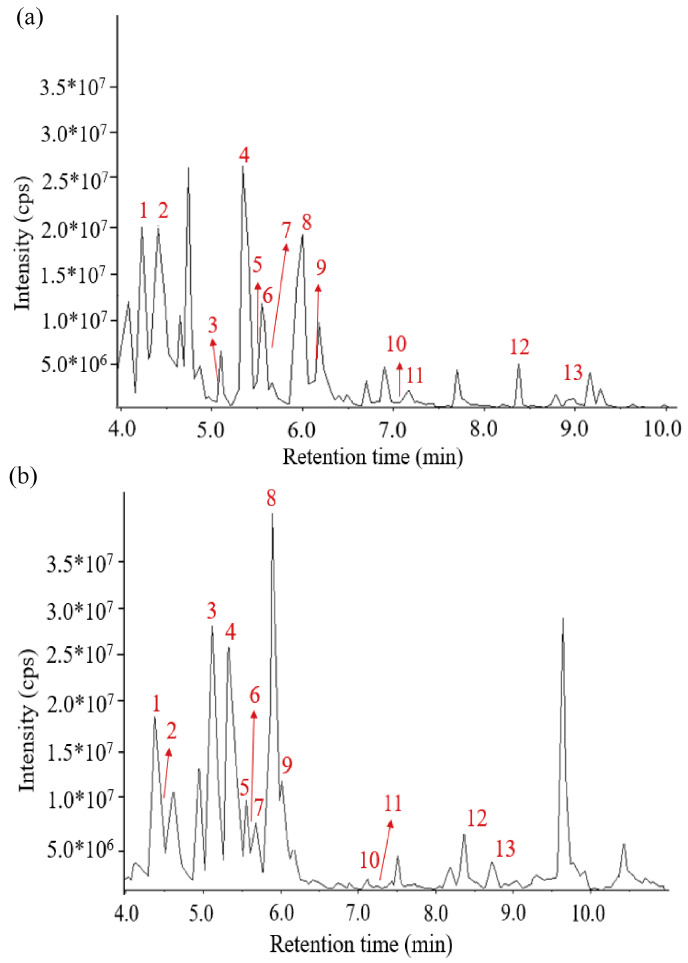
LC–MS chromatogram of (**a**) total ion current-N and (**b**) total ion current-P of 13 ginsenosides.

Based on UPLC-QTOF-MS/MS, 13 kinds of AGGs, namely, Ginsenoside Re (1), Ginsenoside Rg1 (2), Ginsenoside Rf (3), Ginsenoside Rb1 (4), Malonyl Ginsenoside Rc* (5), Ginsenoside Rg2 (6), Malonyl Ginsenoside Rb2* (7), Ginsenoside Rd (8), 6″-Acetyl-ginsenoside Rd (9), Ginsenoside F2 (10), 20(S)-Ginsenoside Rg3 (11), Ginsenoside Rk1 (12), and Ginsenoside Rb1 (13), were identified, as shown in Figure 1 and Table 1. Then, the AGGs were further verified using HPLC and their standard substances. It was found that Rg1 (2), Re (1), Rb1 (4), Rc (5), Rb2 (7), Rd (8), Rf (3), Rg2 (6), Rg3 (10), and F2 (9) could be confirmed, as shown in Figure S3. Of these, compounds 1–10 (Figure S3), considered typical representatives of AGGs in *P. quinquefolius*, have been reported before [22].

### 3.2. In Vitro Antioxidant Activity Analysis of AGGs

**Figure 2:**
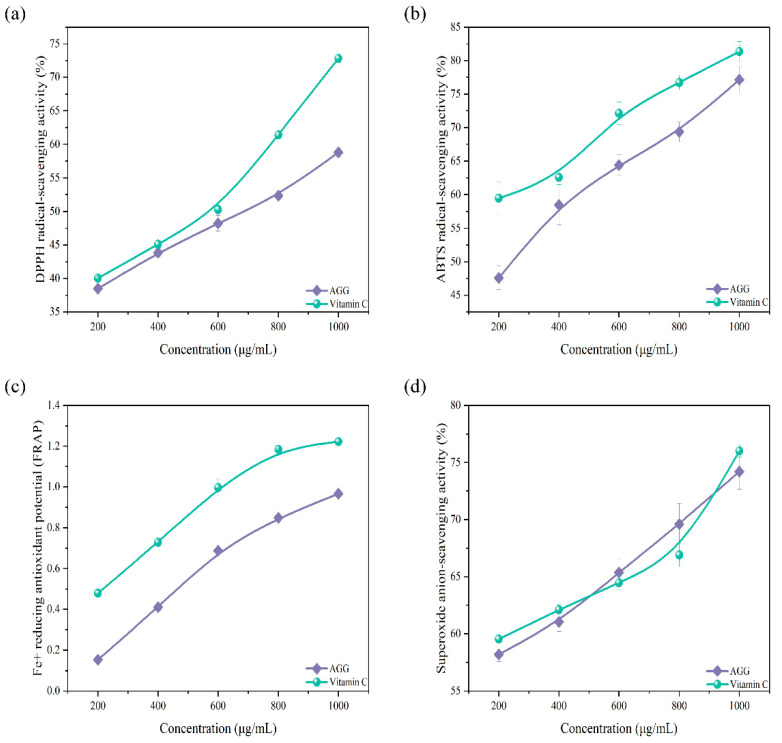
Antioxidant effect analysis of American ginseng in vitro. (**a**) DPPH-radical-scavenging activity, (**b**) ABTS-radical-scavenging activity, (**c**) ferric-reducing antioxidant potential, and (**d**) superoxide-anion-scavenging activity.

In daily life, antioxidants can effectively enhance the body’s immune response and, in addition, can also effectively lower the likelihood of cancer, respiratory tract infections, and cardiovascular and cerebrovascular diseases [23]. Natural antioxidants, including flavonoids, terpenoids, phenols, tannins, and other similar substances, have been widely found in plants [24]. The sample prepared in this work was rich in abundant terpenoids; thus, it was expected to serve as a potential antioxidant. The capacity of AGGs to scavenge free radicals was evaluated as an indication of their antioxidant ability. As shown in Figure 2, AGGs showed significant antioxidant potential by effectively scavenging free radicals, including DPPH, ABTS, and the superoxide anion, in a manner dependent on their concentration. The capacity of AGGs to scavenge DPPH, ABTS, and the superoxide anion increased from 38.5 ± 0.82%, 47.60 ± 1.76%, and 58.20 ± 0.61% at an AGG concentration of 200 μg/mL to 58.8 ± 0.66%, 77.12 ± 1.94%, and 74.2 ± 1.55% at an AGG concentration of 1000 μg/mL, respectively. It was found that the DPPH- and ABTS-radical-scavenging capacity of AGGs was inferior to that of V_C_, whereas the scavenging potential of AGGs for the superoxide radical was similar to that of V_C_. Thus, AGGs showed a noticeable scavenging potential for these radicals. In addition to the free-radical-scavenging capacity, the ferric-reducing antioxidant capacity was also employed to assess the antioxidant potential of AGGs. According to the data presented in Figure 2c, the reducing power exhibited an upward trend as the sample concentrations increased from 200 to 1000 μg/mL.

Previous studies have demonstrated that terpenoids in plants could act as antioxidants [25]. Furthermore, the antioxidant activity of ginsenosides includes not only scavenging activity on the superoxide anion and DPPH and ABTS radicals but also a strong total antioxidant capacity [26]. In addition, ginsenosides play an important role in human daily life. For example, previous reports suggest that ginsenosides may participate in Nrf2-ARE (NF-E2-related factor 2–antioxidant-responsive element)-mediated antioxidant stress pathways, thereby contributing to cancer prevention [26]. Similarly, another study showed that the cardioprotective effect of Ginsenoside Rg1 during myocardial hypoxia and reoxygenation is attributed, at least in part, to its antioxidant activity and ability to maintain intracellular calcium homeostasis [27], and others also inferred that Ginsenoside Rb1 is protective against 6-hydroxydopamine-induced oxidative stress and may enhance heme oxygenase 1 (HO-2) expression via an Nrf1-dependent pathway; a previous study also suggested the potential of Ginsenosides Rb1, Rg1, and 20S for antioxidant activity, the transcriptional activation of the ARE, and synergistic activity, and the Nrf2-ARE-mediated antioxidant pathway may play a role in the overall antioxidative stress activity, which may be important for the health-beneficial effects of ginseng, such as cancer chemopreventive activity [26]. Consequently, this work offers evidence that AGGs can serve as functional foods to decrease the risk of oxidative-stress-related disorders.

### 3.3. Protective Effect of AGGs on H_2_O_2_-Induced Oxidative Injury in RAW 264.7 Macrophages

Prior to assessing the bioactivity of AGGs, it is essential to conduct a toxicological evaluation to ensure their safety. Hence, the influence of AGGs on the viability of RAW 264.7 macrophages was investigated. As depicted in Figure S2, the results indicated that cell viability remained at approximately 100% with the tested concentrations of AGGs, spanning from 50 to 800 μg/mL. Nevertheless, at concentrations exceeding 800 μg/mL, cell viability exhibited a notable reduction (*p* < 0.05). Therefore, AGGs with concentrations lower than 800 μg/mL exhibit limited cytotoxicity in RAW 264.7 macrophages.

**Figure 3:**
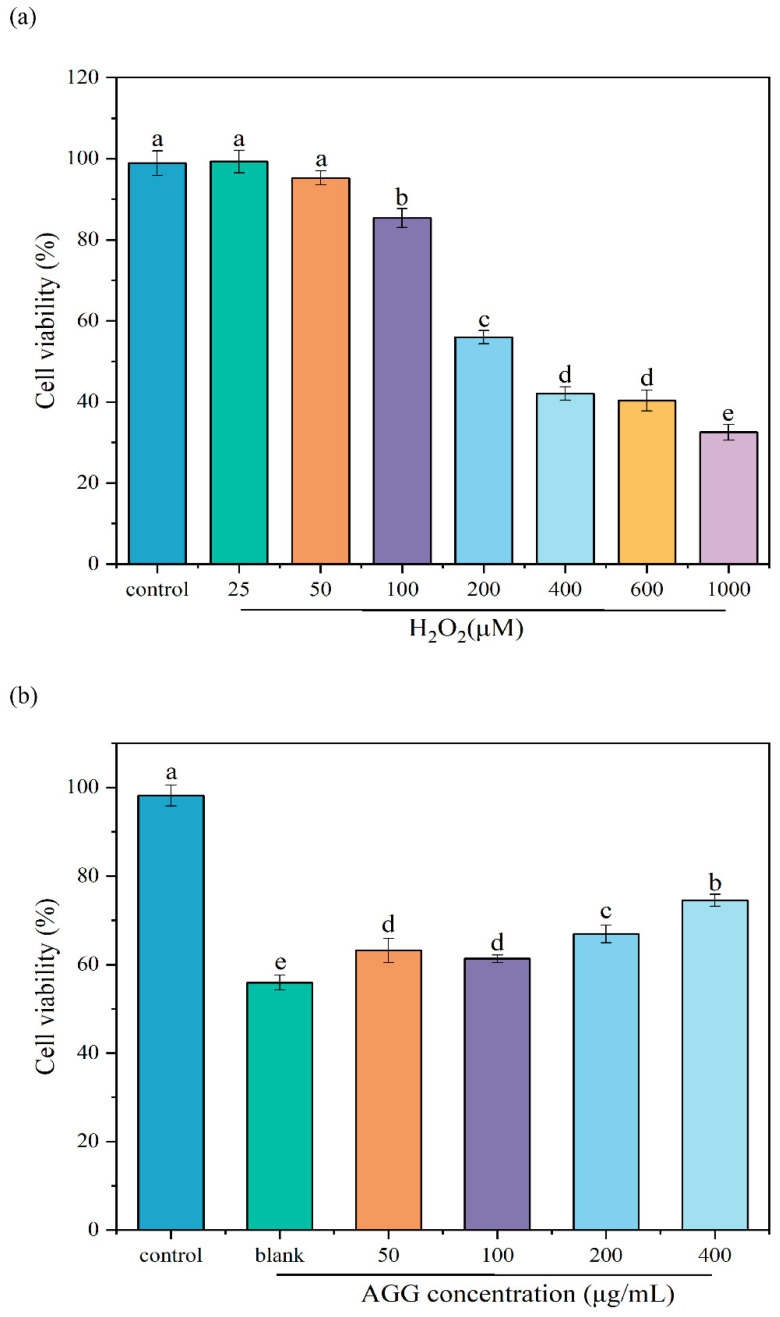
Effects of (**a**) different concentrations of H_2_O_2_ and (**b**) AGGs on cell viability of RAW 264.7 macrophages induced by H_2_O_2_. Different letters (a–e) mean significantly different at *p* < 0.05. Note: control: without-H_2_O_2_ treatment group; blank: group treated with only 200 μM H_2_O_2_.

Extensive research has shown that oxidative stress is related to diverse diseases, making the prevention of oxidative stress a crucial bioactivity for functional foods. The H_2_O_2_-induced oxidative injury model in RAW 264.7 macrophages has recently become a well-established and commonly used in vitro model for evaluating antioxidant activities [28]. In this work, the protective effect of AGGs on H_2_O_2_-induced oxidative injury in RAW 264.7 macrophages was investigated (Figure 3). Firstly, to create a cellular model of oxidative injury induced by H_2_O_2_, the effect of various H_2_O_2_ concentrations on the cell viability of RAW 264.7 macrophages was examined. It was observed that the percentage of cell viability decreased from 98.91 ± 2.80% to 55.97 ± 1.54% upon treatment with 200 μM of H_2_O_2_, which was used for subsequent experiments. As anticipated, pretreatment with AGGs at concentrations ranging from 50 to 400 μg/mL exhibited a dose-dependent increase in cell viability, indicating the protective effect of AGGs against H_2_O_2_-induced oxidative injury.

**Figure 4:**
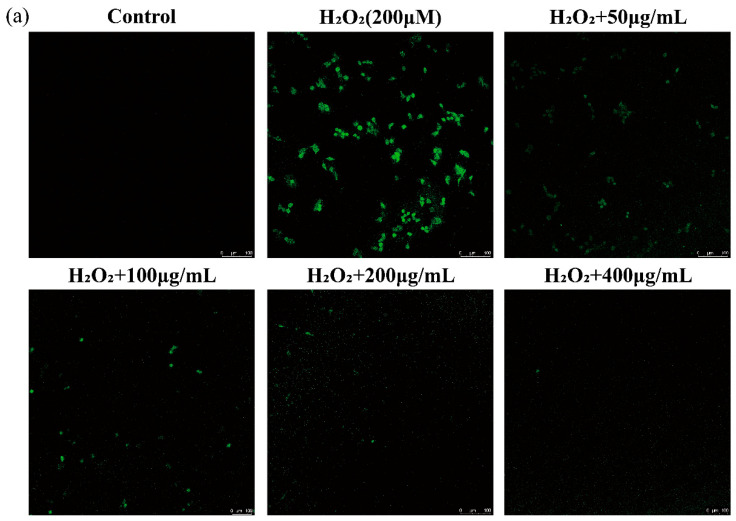

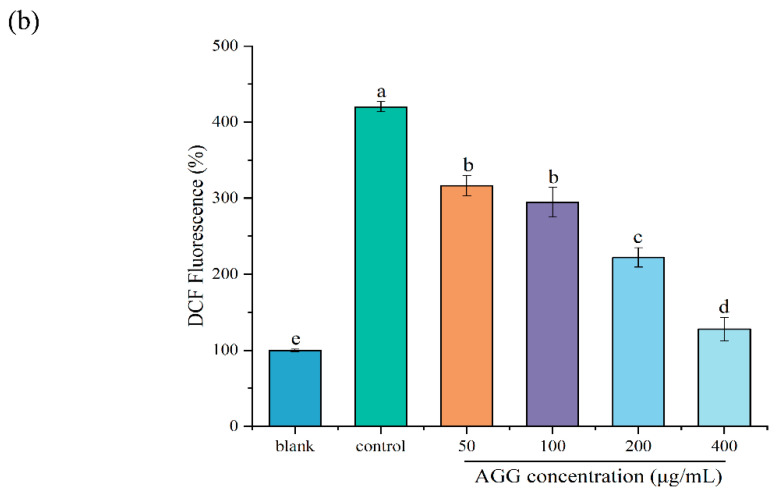
Effects of the extracts on intracellular ROS in LPS-induced RAW 264.7 macrophages. (**a**) Assessment of ROS levels by confocal laser scanning microscopy with DCFH-DA fluorescent dye. ROS levels were evaluated by confocal laser scanning microscopy with DCFH-DA fluorescent dye. Green: DCF fluorescent representation of intracellular ROS. Scale: 100 μm. (**b**) Quantitative analysis of DCF fluorescence intensity percentage in bar graphs. Different letters (a–e) mean significantly different at *p* < 0.05. Note: control: without-H_2_O_2_ treatment group; blank: group treated with only 200 μM H_2_O_2_.

H_2_O_2_, which is produced as a result of dopamine oxidation and enzymatic activity, can lead to oxidative stress and neuronal damage in cell lines by releasing ROS [29]. Thus, the scavenging of ROS by functional foods is a key mechanism for preventing oxidative injury and thereby improving human health. The DCFH-DA probe does not fluoresce on its own but can penetrate cells and be rapidly oxidized by intracellular ROS to produce DCF with green fluorescence [30]. Therefore, the intensity of DCF fluorescence can indirectly serve as a measure of the intracellular ROS level, which was used to detect intracellular ROS in RAW 264.7 macrophages in this study. As depicted in Figure 4a, the level of intracellular ROS detected using laser confocal scanning microscopy significantly increased after treatment with 200 μM H_2_O_2_ compared with that in the control group. As expected, AGG treatment reduced the level of intracellular ROS, and this effect was more pronounced with increasing AGG concentrations. It has been reported that fermented black ginseng protects HepG2 cells from oxidative stress by inducing antioxidant enzyme activity [31]. The present work further demonstrates that AGGs might be important active components of *Panax quinquefolius*, contributing to its antioxidant activity by scavenging ROS. A growing amount of evidence suggests that elevated ROS levels are related to diverse health issues, such as obesity, inflammation, type 2 diabetes (T2D), and cardiovascular disease (CVD); thus, AGG intervention could be expected as a promising approach to improve human well-being and mitigate ROS-related conditions [32].

### 3.4. Anti-Inflammatory Activity of AGGs

The excessive production of proinflammatory factors has been widely reported to be associated with the onset and progression of health issues, including obesity, inflammatory bowel disease, and cancer. The use of drugs for inflammatory conditions in the human body inevitably leads to side effects, which is why herbal medicine is gaining traction as a significant contributor to the pharmaceutical industry, serving as a viable source of medicine [33]. Extensive research has reported that ginsenosides derived from plants have strong anti-inflammatory effects. For example, Ginsenoside Rg1 may improve inflammatory conditions, such as colitis [34]. Likewise, Rb1 and Rb2 extracted from ginseng root could exert an inhibitory effect on TNF-α production in macrophages [35]. Thus, this study focused on evaluating the anti-inflammatory effect of AGGs using a model of LPS-induced RAW 264.7 macrophages.

NO is a transient free radical generated in a wide variety of cells and plays diverse biological roles in response to inflammatory triggers, such as LPS, and is considered a marker of an inflammatory reaction [36]. Thus, the level of NO was measured to evaluate the anti-inflammatory capacity of AGGs. The results showed that the contents of NO significantly increased from 9.67 ± 0.46 μmol/L to 25.87 ± 0.85 μmol/L in the medium of RAW 264.7 macrophages after exposure to 1 µg/mL LPS for 24 h. In contrast, pretreatment with diverse concentrations of AGGs (6.25, 12.5, and 25 µg/mL) led to the remarkable dose-dependent inhibition of NO release in LPS-stimulated RAW 264.7 macrophages. When the concentration of AGGs was 25 µg/mL, the levels of NO in LPS-induced RAW 264.7 macrophages decreased to 19.91 ± 0.11 µmol/L. These findings demonstrate that AGG treatment could lead to a reduction in NO production in cells induced by LPS, showing a concentration-dependent pattern.

**Figure 5:**
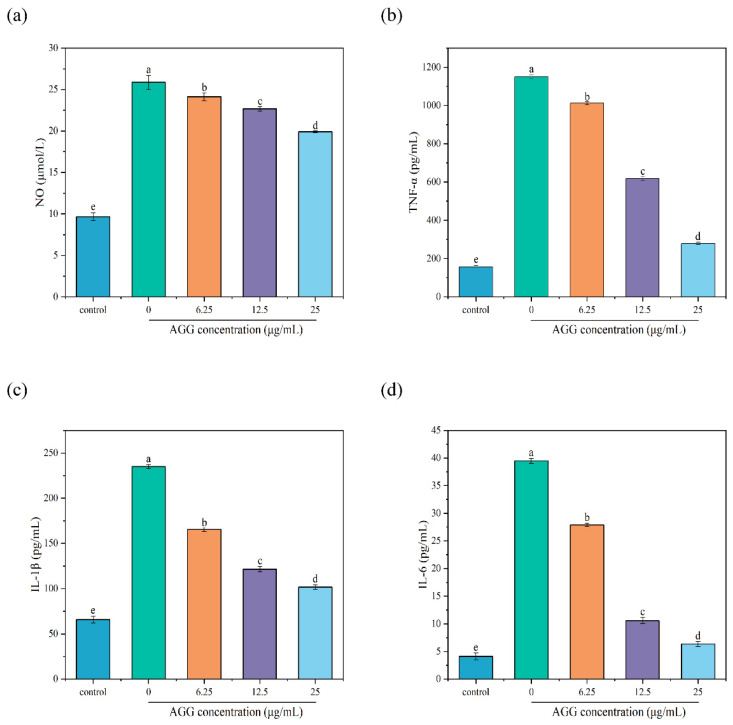
Effects of AGGs on cytokine production of RAW 264.7 macrophages during inflammation. (**a**) NO, (**b**) TNF-α, (**c**) IL-6, and (**d**) IL-1β; different letters (a–e) mean significantly different at *p* < 0.05. Note: control: without-AGG treatment group.

Macrophages are vital components of the human immune system and are present throughout the body, and they play a vital role in the prompt immune reaction against foreign pathogens by releasing proinflammatory mediators, including TNF-α, IL-6, and IL-1β. However, the overexpression of proinflammatory mediators can induce inflammatory reactions and thereby lead to various diseases [37]. Therefore, it is crucial to maintain the balance of inflammation levels for human health. In the present work, the anti-inflammatory potential of AGGs was further evaluated by investigating their modulation of the levels of proinflammatory cytokines, including TNF-α, IL-1β, and IL-6. In Figure 5, it can be observed that the contents of TNF-α, IL-1β, and IL-6 in RAW 264.7 macrophages exhibited a significant increase from baseline levels of 157.5 ± 5.86, 65.82 ± 3.67, and 4.13 ± 0.62 pg/mL to 1150 ± 11.26, 234.94 ± 2.19, and 39.47 ± 0.46 pg/mL after being treated with LPS at a concentration of 1 µg/mL for 24 h, respectively. As expected, pretreatment of RAW 264.7 macrophages with AGGs prior to LPS treatment resulted in the significant dose-dependent inhibition of TNF-α, IL-1β, and IL-6 production. Specifically, at the tested concentration of 25 µg/mL, AGGs showed notable reductions in the levels of TNF-α, IL-1β, and IL-6 in RAW 264.7 macrophages induced by LPS to 279.58 ± 6.45, 101.69 ± 2.64, and 6.37 ± 0.46 pg/mL, respectively. The findings from our study suggest that AGGs exhibit promising potential as therapeutic anti-inflammatory compounds. This effectively confirms the previously reported anti-inflammatory effects of certain ginseng compounds, such as Rg1, Rd, Re, and Rd, both in vivo and in vitro. For instance, Rg1 has been shown to exhibit anti-inflammatory potential in an animal model of 2,4,6-trinitrobenzene sulfuric acid (TNBS)-induced colitis by suppressing inflammatory responses regulated through IRAK activation [38]. Similarly, Rd has been found to inhibit the inflammatory response to ischemia by decreasing the expression of iNOS and COX-2 [39]. Re has demonstrated its anti-inflammatory properties by interfering with the binding between LPS and TLR4 expressed on macrophages, as well as the phosphorylation and degradation of IRAK-1 induced by LPS, and subsequently blocking IKK-α phosphorylation, NF-κB activation, and proinflammatory cytokine expression [40].

This was the first study to evaluate the composition and antioxidant and anti-inflammatory potential of AGGs obtained from forest-grown wild *P. quinquefolius*. The results show that forest-grown wild *P. quinquefolius* has a similar AGG composition to that of *P. quinquefolius* cultivated in farmland [41]. Furthermore, AGGs from the forest-grown wild *P. quinquefolius* showed significant antioxidant and anti-inflammatory activities. Thus, cultivation in the forest is a promising method for increasing the production of *P. quinquefolius*. However, the specific AGGs from forest-grown wild *P. quinquefolius* responsible for antioxidant and anti-inflammatory activities are still unknown. Furthermore, the bioactivities of AGGs should be further investigated with animal models, which will be our next task.

It is essential to understand the bioavailability and pharmacokinetics of ginsenosides to evaluate their therapeutic potential. Previous studies on ginsenoside bioavailability have encountered challenges due to their low oral bioavailability [42]. Factors such as their large molecular size, poor water solubility, and susceptibility to degradation in the gastrointestinal tract significantly affect their absorption [43]. Furthermore, pharmacokinetic studies have shed light on the intricate metabolic fate of ginsenosides. They undergo extensive biotransformation, primarily in the gut and liver, resulting in the formation of various metabolites with modified pharmacological activities [44]. The biotransformation pathways involve processes such as deglycosylation, hydrolysis, and microbial metabolism, leading to diverse pharmacokinetic profiles for different ginsenosides [45]. Additionally, previous research has indicated that the composition and ratios of specific ginsenosides play a pivotal role in their pharmacokinetics. Variations in absorption, distribution, metabolism, and excretion profiles have been observed among different ginsenosides, resulting in diverse in vivo bioactivities [46]. However, information about the bioavailability and pharmacokinetics of AGGs in our present work is still limited and will be the focus of our future work.

## 4. Conclusions

In this study, we showed that terpenoids, particularly AGGs, are the main active substances in forest-grown wild American ginseng. AGGs exhibited dose-dependent scavenging and reducing capacities against free radicals. Additionally, AGGs were found to effectively inhibit intracellular ROS levels in H_2_O_2_-induced RAW 264.7 macrophages, thereby reducing oxidative injury. Furthermore, AGGs could decrease the levels of NO, TNF-α, IL-1β, and IL-6, which showed notable anti-inflammatory activity. Overall, our findings suggest that forest-grown wild American ginseng has the potential to exhibit antioxidant and anti-inflammatory activities, thereby providing a solid theoretical and experimental basis for exploring its potential applications, including the development of dietary supplements, health-promoting beverages, or enriched food products.

## Figures and Tables

**Table 1:** Identification of AGGs.

No.	Molecular Formula	Q1 (Da)	Q3 (Da)	Retention Time	Component Name
1	C_48_H_82_O_18_	945.54	783.49	4.37	Ginsenoside Re
2	C_42_H_72_O_14_	799.48	637.43	4.46	Ginsenoside Rg1
3	C_42_H_72_O_14_	639.45	441.37	5.06	Ginsenoside Rf
4	C_54_H_92_O_23_	1109.61	325.11	5.37	Ginsenoside Rb1
5	C_56_H_92_O_25_	1165.60	411.11	5.53	Malonyl Ginsenoside Rc*
6	C_42_H_72_O_13_	783.49	475.38	5.62	Ginsenoside Rg2
7	C_56_H_92_O_25_	1165.60	411.11	5.66	Malonyl Ginsenoside Rb2*
8	C_48_H_82_O_18_	947.56	325.11	5.93	Ginsenoside Rd
9	C_50_H_84_O_19_	987.55	945.54	6.01	6″-Acetyl-ginsenoside Rd
10	C_42_H_72_O_13_	783.49	621.44	7.05	Ginsenoside F2
11	C_42_H_72_O_13_	783.49	459.38	7.38	20(S)-Ginsenoside Rg3
12	C_42_H_70_O_12_	765.48	603.43	8.50	Ginsenoside Rk1
13	C_54_H_92_O_23_	1109.61	325.11	8.94	Ginsenoside Rb1

## Data Availability

The data used to support the findings of this study can be made available by the corresponding author upon request.

## Supplementary Materials

The following supporting information can be downloaded at https://www.mdpi.com/article/10.3390/foods12234316/s1. Figure S1. The category and percentage of terpenoids in the root extracts of forest-grown wild American ginseng. Figure S2. Effect of AGG on cell viability of RAW 264.7 macrophages. Figure S3. HPLC chromatogram of (a) 10 kinds of AGG standard and (b) sample. Figure S4. Dose-dependent cytotoxicity and IC_50_ values.

## Abbreviations

AGGs	American ginseng ginsenosides
FRAP	Ferric-reducing antioxidant potential
TPTZ	Tripyridyltriazine
ABTS	Aiammonium salt
PMS	Phenazine methosulphate
DPPH	1,1-Diphenyl-2-picrylhydrazyl
NBT	Nitroblue tetrazolium
CCK8	Cell Counting Kit-8
NO	Nitric oxide
ROS	Reactive oxygen species
TNF-α	Tumor necrosis factor-alpha
IL-1β	Interleukin-1 beta
IL-6	Interleukin-6
Vc	Ascorbic acid
