# Supplementary material for: Characterization and In Vitro Antioxidant and Anti-Inflammatory Activities of Ginsenosides Extracted from Forest-Grown Wild Panax quinquefolius L
Source: Foods. 2023 Nov 29;12(23):4316. doi: 10.3390/foods12234316 (PMC10706846; doi:10.3390/foods12234316)
Supplement: Supplementary file 1 [file foods-12-04316-s001.zip › foods-2721274-supplementary.pdf]

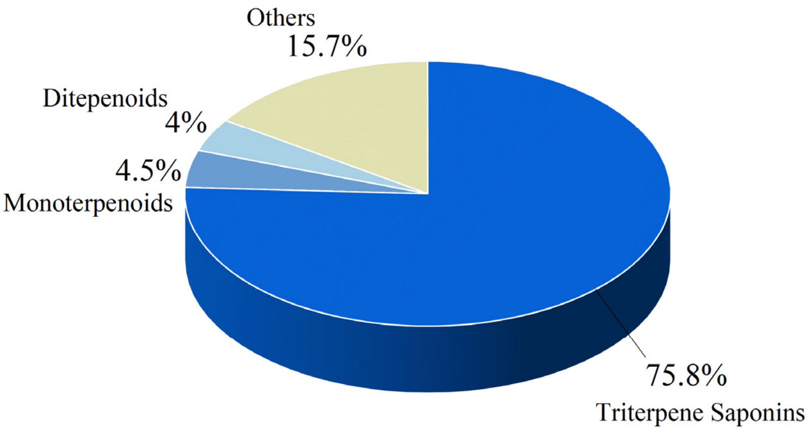

Fig. S1 The category and percentage of terpenoids in the root extracts of forest-grown wild American ginseng.

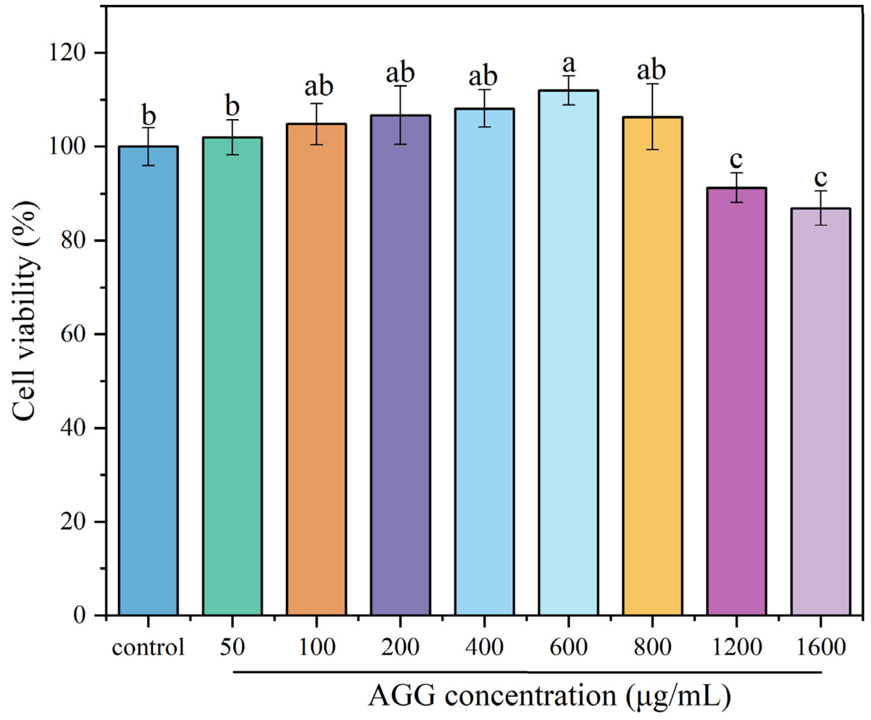

Fig. S2. Effect of AGG on cell viability of RAW 264.7 macrophages. Values are presented as means ± SD (n = 6), a–c with different letter means significantly different at $p < 0.05$. Control: without AGG treatment group.

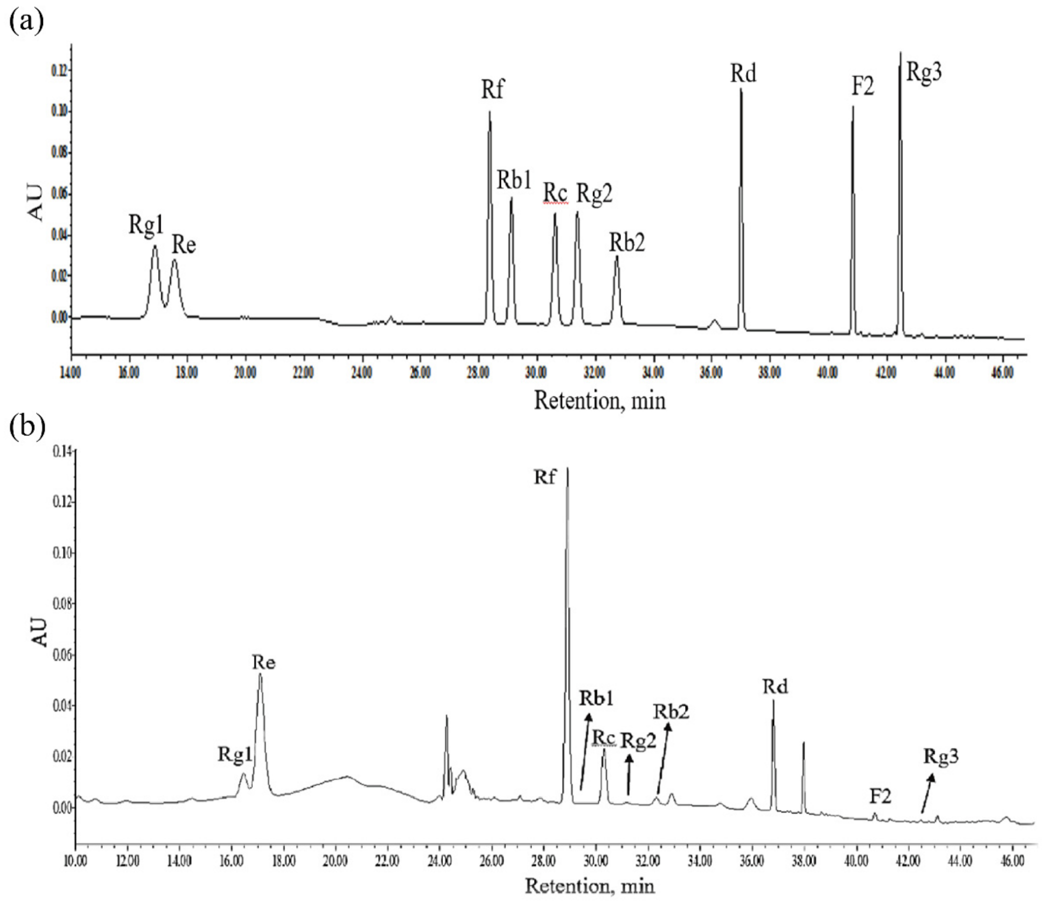

Fig. S3 HPLC chromatogram of (a) 10 kinds of AGG standard and (b) sample

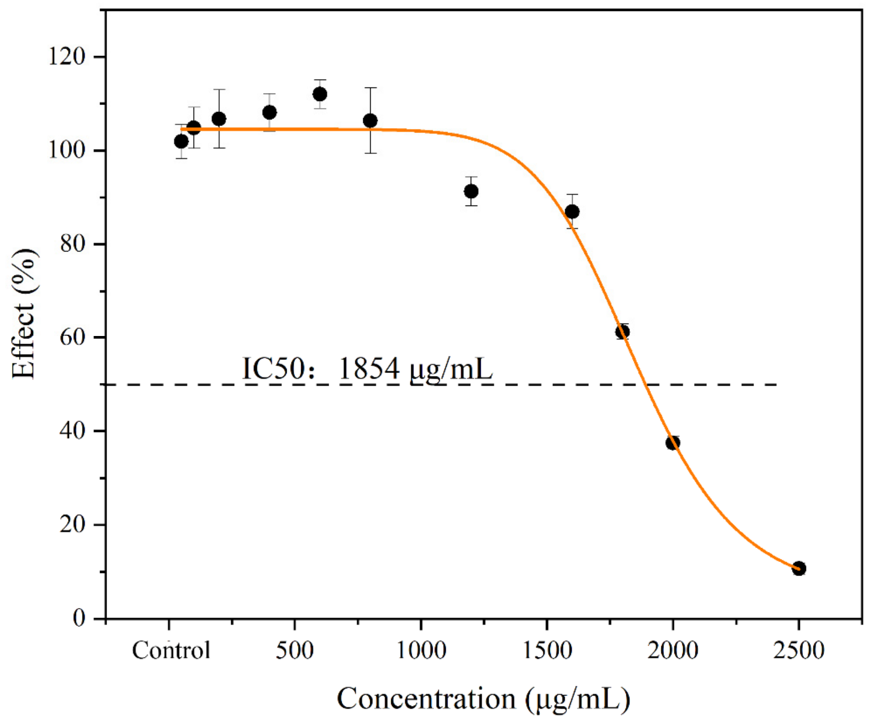

Fig. S4 Dose-dependent cytotoxicity and IC50 values.
